# Construction of a logical model of the line of care for people with chronic kidney disease

**DOI:** 10.11606/s1518-8787.2023057004401

**Published:** 2023-03-15

**Authors:** Liliane Cristina Nakata, Aline Fiori dos Santos Feltrin, Janise Braga Barros Ferreira

**Affiliations:** I Universidade de São Paulo Faculdade de Medicina de Ribeirão Preto Programa de Pós-Graduação em Saúde Pública Ribeirão Preto SP Brasil Universidade de São Paulo. Faculdade de Medicina de Ribeirão Preto. Programa de Pós-Graduação em Saúde Pública. Ribeirão Preto, SP, Brasil; II Universidade de São Paulo Faculdade de Medicina de Ribeirão Preto Departamento de Medicina Social Ribeirão Preto SP Brasil Universidade de São Paulo. Faculdade de Medicina de Ribeirão Preto. Departamento de Medicina Social. Ribeirão Preto, SP, Brasil

**Keywords:** Renal Insufficiency, Chronic, therapy, Disease Management, Healthcare Models, Outcome and Process Assessment, Health Care

## Abstract

**OBJECTIVE:**

To build and validate a logical model of the line of care for people with chronic kidney disease.

**METHODS:**

This is a descriptive study with a qualitative approach, with documentary research and analysis of primary data collected in interviews with key informants, carried out from May to September 2019, in the Guarani Aquifer Health Region, belonging to the Regional Health Department 13. Based on the theoretical framework proposed by McLaughlin and Jordan, five stages were followed: collection of relevant information; description of the problem and context; defining the elements of the logical model; construction and validation.

**RESULTS:**

The logical model was organized into three care dimensions – primary health care, specialized care and high complexity care – composed of structure, process and result components.

**CONCLUSION:**

The constructed logical model has the potential to contribute to the assessment of the line of care for people with chronic kidney disease, in order to achieve better results in the management of this disease, something that favors both the patient and the health system.

## INTRODUCTION

Chronic kidney disease (CKD) is recognized as one of the main public health problems in the world, with an estimated global prevalence of 13.4%^1^. The worldwide increase in this disease is mainly driven by the increase in the prevalence of diabetes mellitus, arterial hypertension, obesity and aging^1^. In Brazil, the estimated prevalence of CKD – in stages 3 to 5 – in adults is 6.7%, and 21.4% in people over 60 years of age^2^.

Between 2000 and 2012, approximately 280 thousand patients were identified in dialysis programs in the Unified Health System (SUS) network, which corresponded to 85% of dialysis performed in the country^3^. In 2015, expenses with renal replacement therapy (RRT) represented more than 2 billion *reais*, corresponding to 5% of SUS expenses with medium and high complexity services, consumed by the partial management of a single disease. Furthermore, its incidence is increasing, which ratifies prevention as an action of interest and importance for public health, highlighting the role of primary health care (PHC)^4^.

In 2017, there were 1.2 million deaths due to CKD, placing it in the 12th position in causes of death in the world, while, in Brazil, this disease was responsible for 35,000 deaths, occupying the 10th position^5^.

Although the prevalence of CKD is high, the first guideline for its diagnosis and treatment was only issued in 2002, by the National Kidney Foundation, in the document *Kidney Disease Outcomes Quality Initiative *(K/DOQI)^6^. In Brazil, the National Policy for Attention to Patients with Kidney Disease (PNAPDR) was only published in 2004^7^.

In 2014, the clinical guidelines for the care of patients with CKD in the SUS and the criteria for organizing the line of care for people with CKD were published – contained in the health care network (HCN) for people with chronic diseases^8,9^. In order to organize the care network and the financing of actions related to the approach to CKD, the line of care presents the following attributions of the HCN care points, according to the components: PHC and specialized outpatient care, the latter subdivided into Specialized Unit in CKD, High Complexity Care Unit in Nephrology and Specialized Unit in CKD with RRT/Dialysis^9^. These guidelines marked a step forward in the country’s public policy, since they systematized this line of care based on comprehensiveness – mainly with PHC – and defining early diagnosis and timely treatment of CKD as one of the attributions of its team.

Thus, there are legal subsidies that favor changes in the work processes of the PHC teams and, also, that establish quality indicators for the monitoring and evaluation of care for people with CKD^10^. In this sense, a strategy that can contribute to this evaluation is the development of a logical model, as it allows visually and systematically presenting the relationships between the necessary resources, interventions and effects – products, results and impact – that a program/intervention intends to achieve^11,12^.

Health evaluation has the potential to present logical arguments of how and why a program is or is not meeting the specific needs for which it was created. In turn, the logical model presents itself as an evaluative tool by detailing characteristics of a program, establishing the logical relationship between its components and the expected results in the short, medium and long term^12^. Modeling an intervention makes it possible to explain the links between the intervention and its effects, through a schematic representation that reveals its structure, processes and results. Modeling must, therefore, explain the logical path of the intervention and reveal its objectives and, for this reason, it must be done in interaction with the actors who operate it, making it possible to improve the intelligibility of a complex system^13^. Among the advantages of using the logical model are: building a common understanding of the program and resource expectations, the number of customers reached and their results; its usefulness for program design or improvement; presenting the program’s place within the organization, and presenting a balanced set of key performance measurement and evaluation questions that improve data collection and the program’s usefulness^12^.

Thus, the objectives of this study were to develop and validate the logical model of the line of care for CKD patients.

## METHODS

In order to build and validate the logical model, a descriptive study with a qualitative approach was carried out, from May to September 2019, in the Guarani Aquifer Health Region – which comprises 10 municipalities, with a total of 945,738 inhabitants –, belonging to the Regional Health Department (DRS) 13 – Ribeirão Preto-SP^14^. The line of care for people with CKD for this Health Region was drawn up by a multidisciplinary working group representing all parts of the HCN, but its monitoring had not yet started. There was, then, a manifestation of the DRS 13 to the researchers about the interest in carrying out an investigation that would help the evaluation process, considering the worrying rates of this Region regarding the prevalence of CKD. Finally, this condition justified the choice of this field of study.

The logical model was built according to the five stages proposed by McLaughlin and Jordan^12^: 1) Collecting relevant information; 2) Describing the problem and context; 3) Defining the elements of the logical model; 4) Building the logical model; and 5) Validating the logical model.

In the development of steps 1, 2, 3 and 4, documentary research was carried out. The criteria used to select the documents were: 1) authenticity, that is, the document is of unquestionable origin; 2) credibility, it is an original and undistorted document; 3) representativeness, within its typology – in this case, legal documents –, which present the content to be analyzed; and 4) meaning, that is, whether the document was clear and comprehensible^15^.

In stages 1 and 2, the documentary research sought to understand the theory and legislation at the federal, state and regional levels of the SUS, which supported the development of the line of care for people with CKD; the survey of information on the estimate of the number of cases in the different stages of CKD – IBGE population estimate –, the parameters of Ordinance GM/MS no. 1.631/GM/2015^16^, and the identification of the prevalence rate of patients on dialysis in the Guarani Aquifer Health Region. At this stage, the primary information obtained from the interviewees was also considered (Box).

**Box:** Script for the semi-structured interview conducted with key informants to identify relevant contextual factors and validate the Logical Model.

Guiding question script	
Identification of the context of the preparation and implementation of the LC of the person with CKD	1) When did the construction of the LC of the person with CKD start? What determined the beginning of this work? 2) Who are the actors who participated in this process? How did it happen? 3) What are the steps after the preparation of the LC? 4) After the preparation and other steps for the approval of the LC, what actions were triggered? 5) What are the main factors that determine the effective implementation of the LC in your opinion? Why? 6) How was the selection of indicators for monitoring and evaluating the LC carried out? Where is the data for such indicators obtained? 8) Who is the regional steering group? Was a follow-up form prepared to monitor compliance with the follow-up protocols for patients provided for in the LC?
Validation of the logical model^12^	1) In the analysis of the logical model prepared, based on the analyzed documents, what observations do you have to make? 2) Is the level of detail sufficient to create understandings of the elements and their interrelationships? 3) Is the program logic complete? 4) Is the program logic theoretically consistent, that is, do all elements logically fit together? 5) Are there other plausible ways to achieve the results of the program?

LC: line of care; CKD: chronic kidney disease

In the development of steps 3 and 4, the documents analyzed were: the PNAPDR^7^, the guidelines for organizing the HCN and for the Care of CKD Patients in the SUS^17,18^, the criteria for organizing the line of care for people with CKD^9^, the Instructional Guide for local organization of the line of care for people with CKD^19^, the regulations on the care of people with CKD^20^, and the line of care for people with CKD of the HCN for people with chronic diseases of DRS 13. Information about this line of care was organized in the logical model, according to the care dimensions – PHC, specialized care and high complexity care –, in the form of a color diagram. Each color represented, in the rows, the care dimensions and, in the columns, the components of the “line of care” intervention: inputs/resources, actions/activities, product, intermediate result and final result/impact.

The initial model was appreciated by three health professionals: a nephrologist, Master in Health Sciences and PhD student in Public Health, and two nurses working in PHC, a Master and the other a PhD in Public Health. These professionals, chosen intentionally, contributed to checking the components of the logical model and complementing the information, characterizing it as a preliminary analysis. After that, step 5 began, carried out in person by one of the researchers and which aimed to present to the key informants the elements of the logical model, its concepts and its purpose – before the validation process – and, subsequently, to verify whether the proposed components and organization represented the logic of the line of care for people with CKD.

The logical model was validated by six key informants, intentionally selected: a member of the planning team of a municipality in DRS 13, a PHC coordinator from the Guarani Aquifer Health Region, a nurse from a dialysis service, two professionals from the management/planning team of DRS 13 – these professionals who participated in the preparation of the line of care for people with CKD of DRS13 –, and a professional from the management/planning team of the São Paulo State Department of Health (SES -SP), also coordinator of the non-communicable chronic diseases (NCDs) steering group. Finally, a semi-structured interview was carried out with key informants, following the questions pointed out by MClauglin and Jordan^12^ (Box), and the study was approved by the Research Ethics Committee (CAAE: 58545116.3.000.5414).

## RESULTS

### Context Characterization

CKD is recognized as a complex disease that requires multiple approaches in its treatment. It is even associated with high morbidity and mortality rates, prevalence and incidence rates still unknown in many countries, and a great socioeconomic impact, becoming a challenge for global public health^21^. In Brazil, according to the National Health Survey (PNS, 2013), the prevalence of self-reported CKD is 1.42%, that is, approximately two million individuals, which reveals the dimension of the disease in the country^21^. Early diagnosis, immediate referral, and implementation of measures to reduce/stop the progression of CKD are among the key strategies to improve its outcomes^21^.

The PNAPDR proposed the organization of the comprehensive care line – promotion, prevention, treatment and recovery –, permeating all points of care, such as expanding the coverage of care for patients with arterial hypertension and diabetes mellitus, the qualification of assistance and the promotion of continuing education for health professionals^7^.

In 2010, the ordinance that established the guidelines for the organization of HCN in SUS was published; it aimed to promote the systemic integration of health actions and services, with the provision of continuous, comprehensive, quality, responsible and humanized care, in addition to increasing SUS’s performance in terms of access, equity, clinical and health efficacy and economic efficiency^17^. This regulation highlighted that, although the advances were representative, the fragmentation of health actions and the need to qualify care management was still evident. Thus, the development of HCN was presented as an innovative organizational process, with the potential to positively impact health indicators.

In this logic, in the years 2013 and 2014, the HCN of People with NCDs was established, which established guidelines for the organization of its Care Lines, for its principles and objectives and for the competences of each federal entity. The Care Lines must express the assistance flows that must be guaranteed to the user, in order to meet the health needs related to a chronic condition and define the actions and services that will be offered by each component of the HCN of People with NCDs, based on clinical guidelines and the reality of each Health Region^8^.

In turn, the criteria for organizing the line of care for people with CKD, and the clinical guidelines for care, were published in 2014, defining the attributions of PHC, specialized outpatient care, and high complexity care^18^.

From the publication of the ordinance of the line of care for people with CKD, the health regions began the process of regional discussion about it. According to key informants, a steering group was formed with representatives from the three health regions of the DRS 13 territory, from the hemodialysis service providers and from the DRS 13 planning group. The group met to develop the line of care based on the organization of services, which was approved by the Regional Interagency Commissions (CIR) of the three regions of DRS 13 and by the Bipartite Interagency Commission (Deliberation CIB/SP – 47/2015). However, according to the interviewees, until the date of the interview, in May 2019, there had been no monitoring of the line of care within the PHC scope, while the quality indicators of medium and high complexity services were monitored, since they were related to qualification and remuneration of specialized services^18^.

*“A regional steering group was formed with participants from the municipalities, representatives of management and services, approved in CIR, and we followed the steps set out in the ordinance … The Care Line was approved in the CIR, we await publication by the Ministry of Health which, due to the release of financial resources, took more than two years”*. (Interviewee No. 1)

The estimated number of cases in the different stages of CKD in the Guarani Aquifer Health Region (Table 1) indicates that approximately 79,070 people – 8.36% of the total population – should be followed up by the PHC services in stages 1, 2 or 3 of CKD, while 1,336 people – 0.14% of the total population – would be monitored in specialized services in stages 4 or 5, with or without dialysis. The prevalence of patients on dialysis in this Region has been increasing over the years (Table 2). The difficulty in accessing data from the Health Information Systems (SIS), to confirm these estimates, points to one of the critical nodes of health management and, mainly, those referring to the care provided in PHC. Therefore, the lack of reliable and timely information hinders the monitoring of the line of care and care planning.

**Table 1:** Estimate of the number of cases in the different stages of chronic kidney disease, based on the population estimate from IBGE 2020 and the parameters of Ordinance GM/MS no.. 1.631/GM/2015, RS Guarani Aquifer, RP-SP, 2020.

County	Pop. IBGE 2020	IBGE 2020/DSUS estimate over 20 years	Stage 1 (9.6% pop. > 20 years)	Stage 2 (0.9% pop. > 20 years)	Stage 3 (1.5% pop. > 20 years)	Stage 4 (0.1% pop. >20 years)	Stage 5 New Dialysis (0.014% pop. > 20 years)	Stage 5 Dialysis Prevalence (0.075% pop. > 20 years)	Deaths (0.013% pop. > 20 years)
Cravinhos	35,579	26,083	2,504	23	391	26	4	20	3
Guatapará	7,709	5,482	526	5	82	5	1	4	1
Jardinópolis	44,970	32,018	3,074	28	480	32	4	24	4
Luís Antônio	15,292	10,644	1,022	9	160	11	1	8	1
Ribeirão Preto	711,825	535,826	51,439	463	8,037	536	75	402	70
Santa Rita do Passa Quatro	27,600	21,785	2,091	19	327	22	3	16	3
Santa Rosa de Viterbo	26,753	20,190	1938	17	303	20	3	15	3
São Simão	15,385	11,434	1,098	10	172	11	two	9	1
Serra Azul	14,981	11,819	1,135	10	177	12	two	9	two
Serrana	45,644	31,553	3,029	27	473	32	4	24	4
**Total**	**945,738**	**706,834**	**67,856**	**611**	**10,603**	**707**	**99**	**530**	**92**

IBGE: Brazilian Institute of Geography and Statistics; Datasus: Department of Informatics of the Unified Health System.

**Table 2:** Prevalence rate (per 100,000 inhabitants) of dialysis patients in the Guarani Aquifer Health Region, by municipality, 2010 to 2017.

Health Region/Mun.	2010	2011	2012	2013	2014	2015	2016	2017
Cravinhos	75.8	71.99	68.25	86.09	60.94	72.48	80.91	83.25
Guatapará	129.29	128.41	99.19	112.58	83.86	97.17	96.55	95.93
Jardinópolis	47.88	47.17	51.64	68.69	67.68	74.09	75.68	60.33
Luís Antônio	62.26	51.9	117.79	90	79.58	92.87	129.17	126.81
Ribeirão Preto	67.57	68.43	69.4	71.29	69.82	74.55	77.11	80.06
Santa Rita do Passa Quatro	67.99	52.89	52.91	64.26	68.06	86.98	87.02	87.06
Santa Rosa de Viterbo	75.5	95.74	99.15	106.6	134.27	125.18	128.36	95.63
São Simão	48.81	69.5	103.89	89.74	116.94	95.98	109.3	74.89
Serra Azul	26.75	26.49	26.24	26	68.67	59.51	75.87	91.93
Serrana	48.94	73.61	67.54	61.62	55.87	69.41	82.75	79.4
**Total**	**65.89**	**67.84**	**69.73**	**72.39**	**71.56**	**76.66**	**80.71**	**80.8**

Source: Tabnet, São Paulo State Health Secretariat (SES/SP). Attended patients: SES/SP/SIA-SUS - SUS Outpatient Information System. Population: Estimates - SEADE Foundation. 1. Prevalence rate: No. of patients undergoing renal dialysis treatment in SUS (continuous ambulatory peritoneal dialysis - CAPD, automated peritoneal dialysis - APD, intermittent peritoneal dialysis - IPD, hemodialysis, hemodialysis in patients with human immunodeficiency virus), per 100,000 inhabitants in the resident population.

*“Today what you have in the official systems, for example, the exams carried out, you can pull, in what the Care Line proposes, the exams of each phase that we have to follow. So, as a production, it can be surveyed. What is more complicated, I think, is precisely in relation to Primary Care, how many patients I am following, how many hypertensive patients there are, what stage they are in…”*. (Interviewee No. 6)

*“These hypertensive patients are difficult, we don’t have a tool to assess this. Hiperdia is over. It was good when it was there, you know, and the municipalities used it and it was linked to the distribution of medicine…”*. (Interviewee No. 2)

When asked about the factors they considered to influence the effective implementation of the line of care, the interviewees pointed out conditions related to the work process, care models, professional qualification and the quality of PHC.

*“The factors that make it difficult are the work processes, they (the professionals) do not understand what a network is, each one does a little bit. Truly understanding why there are so many patients is also lacking. When we went to do the Care Line, we did not find much data, the municipalities do not have this data, it was necessary to use references from studies”*. (Interviewee No. 1)

*“I’m critical of the performance. I think we have the right structures, we are in a privileged region in terms of support and technology, but we have a lot to do with the way it is organized, the model of assistance, training and qualification of professionals, I think this hinders”*. (Interviewee No. 2)

*“The specialized *[care] *and discharge work well, they play their role, I think we have to intervene in Primary Care”*. (Interviewee No. 1)

### Preparation and Validation of the Logical Model

The logical model, built using a diagram (Figure), presents the activities and expected results by the HCN care dimension, in addition to identifying its necessary inputs and resources. The definition of each dimension, presented in the model, was based on the criteria for organizing the line of care for people with CKD^9^, which subdivide the guidelines and responsibilities according to the level of technological density of the HCN services. Likewise, the actions that must be performed were based on the activities already foreseen by the SUS norms, and, based on them, the inputs for carrying out the planning as a whole were established. The logical model was improved based on its prior appreciation, by which information was complemented and verified before validation. During validation, the model was presented to six key informants, who participated in the development of the care line for people with CKD or worked at some point in the HCN. The semi-structured questionnaire (Box), proposed by McLaughlin and Jordan^12^, was applied to participants in step 5, who answered affirmatively to the questions. Key informants explained the context in which the line of care was developed and validated the model^12^, not suggesting the inclusion of any new information.

**Figure:**
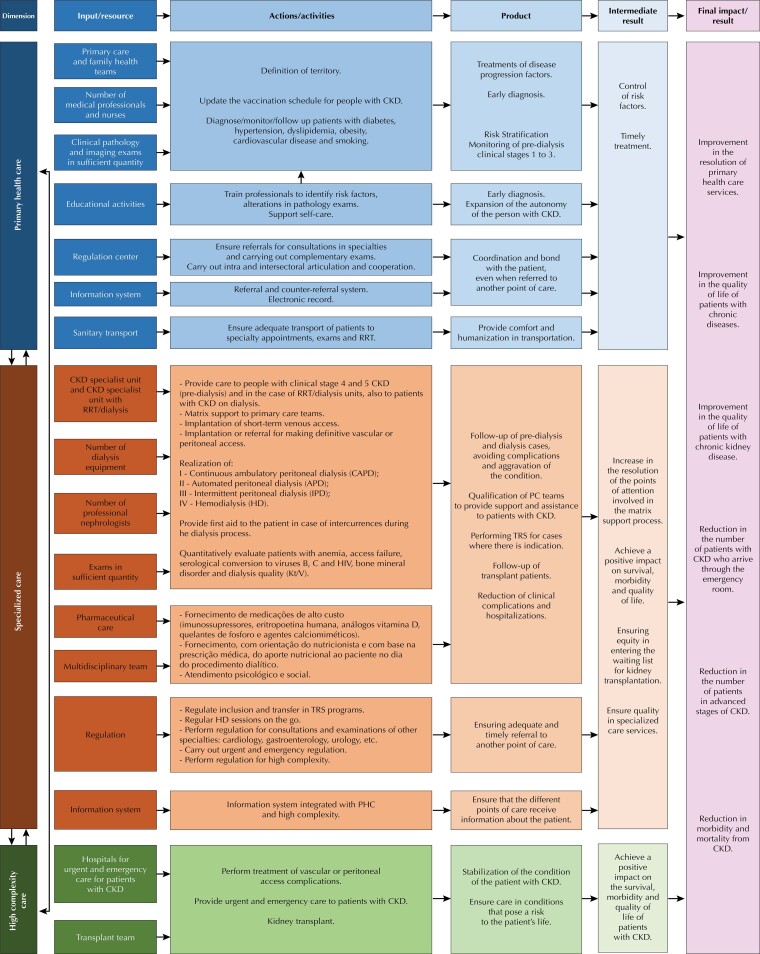
Logical model. CKD: chronic kidney disease; RRT: renal replacement therapy; PHC: primary health care; Kt/V: method that evaluates the adequacy of the patient’s dialysis.

## DISCUSSION

CKD has received attention from the international and national scientific community, and its high prevalence is demonstrated in recent studies^23^, as well as the increase in the estimated incidence rate, which, in 2018, was 20% higher than that observed in 2013. In Brazil, arterial hypertension remains the main underlying cause of CKD, followed by the renal disease caused by diabetes^24^.

SUS has had the PNAPDR since 2004, but it was only almost ten years later, in 2013, that the CKD care line and clinical guidelines were published. However, data from the Brazilian Census of Dialysis, up to 2018, showed a growing increase in the rates of incidence and prevalence of patients on dialysis^24^, indicating the need to strengthen policies to prevent this condition.

The line of care for people with CKD establishes the attributions of the HCN care points, guiding the organization of the work of professionals and services. However, its monitoring and evaluation basically cover indicators aimed at high complexity^19^, ignoring PHC.

In this study, the preparation of the logical model, based on the analysis of official documents, enabled to build a first diagram of the relationship between structure, process and result. The interview with professionals who worked at different points in the care line detailed the components of this model, making it more complete and enabling the identification of factors in the internal context that could interfere with its implementation.

With regard to the care dimensions of the logical model, we highlight the attention to PHC given by key informants. All of them commented on its fundamental role in organizing the line of care, but also on its weaknesses related to the work process, training, team qualification and the lack of information for monitoring and evaluation.

The first block of activities of the PHC dimension encompasses a large part of the actions foreseen by the National Primary Care Policy^25^. In this sense, territorialization is the first stage of planning. Through it, the area in which the health service operates and its socio-environmental conditions is recognized, the population and its health problems are characterized, as well as the dynamics of its interaction with adjacent social equipment and with other sectors, in order to identify needs and propose health promotion and protection actions, in addition to disease prevention^25^.

Therefore, it is essential to guarantee multidisciplinary teams and infrastructure in the territory that allow knowledge of the problems/needs and potential of the community, fundamental conditions to subsidize the planning, monitoring and evaluation of health actions.

In the second block, whose input is the carrying out of educational activities, the absence of popular health education as an action is related to the fact that it is not included in the item “health education”, the norms consulted and the experience of key informants about the theme. However, we understand that it is important for user empowerment and consequent participation in decision-making.

The following blocks are related to the guarantee of care in specialized care, transport and referral and counter-referral system. Regarding the use of electronic medical records, although it could be considered as an input, given the availability of the PEC e-SUS by the Ministry of Health, it was considered as an action, whether the PEC e-SUS itself or any other system of that type. The electronic medical record allows the optimization of clinical care, accessibility to data on care and procedures performed, integration of information, registration of households, in addition to supporting teaching and research. In addition, this record favors communication in the HCN, for example, through a reference and counter-reference system, contributing to the coordination and continuity of care and linking the user to the teams.

Regarding PHC products, although they are difficult to measure, they refer to what is expected from the proposed activities. It is believed that the logical model provokes reflections on them and, consequently, the election of structure, process and result indicators by dimension of the line of care.

The dimension of specialized care was referred to as the part of the care line that “works best”, with well-defined monitoring indicators, as well as high complexity. It is noteworthy that, among the interviewees, there were no highly complex professionals, which may be a limitation of the study, considering that they could propose modifications and/or additions to the model.

The logical model, although it was elaborated in an SR, which may represent a limitation, includes the three dimensions of health care: primary, secondary and tertiary. Its use by professionals and managers, compared with the different realities, can collaborate with the monitoring/evaluation of the line of care, and with the identification of potentialities and challenges, helping in planning and decision-making^26^.

## Final Consideration

There is recognition that, in the country, the approach to patients with CKD in the SUS needs to be improved. The line of care for the person with CKD, when detailing the attributions by the HCN care dimensions, is characterized as a guide for organizing the work process of professionals and services, with emphasis on PHC, a component that is still little explored and valued.

In the context of health assessment, the presented logical model helps in proposing performance indicators applicable to the three care dimensions, but mainly to PHC, because, although it is recognized as a network organizer and care coordinator, it has several organizational weaknesses, and, therefore, it has been underprivileged in discussions and investments. Also, the indicators to be developed from this line of care for the person with NCDs can be used in other Lines of the type, contributing to the improvement of the management action and enabling the effective implementation of this care organization strategy.

It is admitted that the suggested logical model has the potential to contribute to processes of evaluating the line of care for the person with CKD, with a view to achieving better results in the management of this disease, both for the patient and for the health system.
