# Supplementary material for: Construction of a logical model of the line of care for people with chronic kidney disease
Source: Rev Saude Publica. 2023 Mar 15;57:14. doi: 10.11606/s1518-8787.2023057004401 (PMC10118404; doi:10.11606/s1518-8787.2023057004401)
Supplement: Translation

# Translation: Construção de modelo lógico da linha de cuidado da pessoa com doença renal crônica

**Authors:** Liliane Cristina Nakata, Aline Fiori dos Santos Feltrin, Janise Braga Barros Ferreira

**Affiliations:** I Brasil Universidade de São Paulo. Faculdade de Medicina de Ribeirão Preto. Programa de Pós-Graduação em Saúde Pública. Ribeirão Preto, SP, Brasil; II Brasil Universidade de São Paulo. Faculdade de Medicina de Ribeirão Preto. Departamento de Medicina Social. Ribeirão Preto, SP, Brasil

**Keywords:** Insuficiência Renal Crônica, terapia, Gerenciamento Clínico, Modelos de Assistência à Saúde, Avaliação de Processos e Resultados em Cuidados de Saúde

## Abstract

**OBJETIVO:**

Construir e validar um modelo lógico da linha de cuidado da pessoa com doença renal crônica.

**MÉTODOS:**

Trata-se de um estudo de caráter descritivo e com abordagem qualitativa, sendo feitas pesquisa documental e análise de dados primários coletados em entrevistas com informantes-chave, realizadas de maio a setembro de 2019, na Região de Saúde do Aquífero Guarani, pertencente ao Departamento Regional de Saúde 13. A partir do referencial teórico proposto por McLaughlin e Jordan, foram seguidas cinco etapas: a coleta de informações relevantes; a descrição do problema e do contexto; a definição dos elementos do modelo lógico; e a construção e validação.

**RESULTADOS:**

O modelo lógico foi organizado em três dimensões assistenciais – atenção primária à saúde, atenção especializada e atenção de alta complexidade – compostas pelos componentes de estrutura, processo e resultado.

**CONCLUSÃO:**

O modelo lógico construído tem potencial para contribuir com a avaliação da linha de cuidado da pessoa com doença renal crônica, ao visar o alcance de melhores resultados no manejo dessa doença, o que favorece tanto o seu portador quanto o sistema de saúde.

## INTRODUÇÃO

A doença renal crônica (DRC) é reconhecida como um dos principais problemas de saúde pública no mundo, com prevalência global estimada em 13,4%^1^. O aumento mundial dessa doença é impulsionado, principalmente, pelo acréscimo do predomínio da diabetes mellitus, da hipertensão arterial, da obesidade e do envelhecimento^1^. No Brasil, a prevalência estimada de DRC – nos estágios 3 a 5 – em adultos é de 6,7%, sendo de 21,4% em pessoas acima de 60 anos^2^.

Entre os anos de 2000 e 2012, foram identificados aproximadamente 280 mil pacientes em programas de diálise na rede do Sistema Único de Saúde (SUS), o que correspondeu a 85% das diálises realizadas no país^3^. Em 2015, os gastos com terapia renal substitutiva (TRS) representaram mais de 2 bilhões de reais, correspondendo a 5% dos gastos do SUS com serviços de média e alta complexidade, consumidos pelo manejo parcial de uma só doença. Ademais, a sua incidência está aumentando, o que ratifica a prevenção como ação de interesse e importância para a saúde pública, destacando-se o papel da atenção primária à saúde (APS)^4^.

Em 2017, ocorreram 1,2 milhões de óbitos em decorrência da DRC, colocando-a na 12ª posição em causas de morte no mundo, enquanto que, no Brasil, essa doença foi responsável por 35 mil mortes, ocupando a 10ª posição^5^.

Embora a prevalência da DRC seja alta, a primeira diretriz para o seu diagnóstico e tratamento foi realizada apenas em 2002, pela *National Kidney Foundation*, no documento *Kidney Disease Outcomes Quality Initiative* (K/DOQI)^6^. No Brasil, a Política Nacional de Atenção ao Portador de Doença Renal (PNAPDR) só foi publicada em 2004^7^.

Em 2014, foram publicadas as diretrizes clínicas para o cuidado ao paciente com DRC no SUS e os critérios para a organização da linha de cuidado da pessoa com DRC – contida na rede de atenção à saúde (RAS) das pessoas portadoras de doenças crônicas^8,9^. Com o objetivo de organizar a rede assistencial e o financiamento das ações relacionadas à abordagem da DRC, a linha de cuidado apresenta as seguintes atribuições dos pontos de atenção da RAS, segundo os componentes: APS e atenção especializada ambulatorial, esta última subdividida em Unidade Especializada em DRC, Unidade de Assistência de Alta Complexidade em Nefrologia e Unidade Especializada em DRC com TRS/Diálise^9^. Essas diretrizes marcaram um avanço na política pública do país, uma vez que sistematizaram essa linha de cuidado pautando-se na integralidade – principalmente com a APS – e definindo como uma das atribuições de sua equipe o diagnóstico precoce e o tratamento oportuno da DRC.

Assim, nota-se a existência de subsídios legais que favorecem mudanças nos processos de trabalho das equipes de APS e, ainda, que estabelecem indicadores de qualidade para o monitoramento e avaliação da atenção às pessoas com DRC^10^. Nesse sentido, uma estratégia que pode contribuir com essa avaliação é a elaboração do modelo lógico, pois permite apresentar de maneira visual e sistemática as relações entre os recursos necessários, intervenções e efeitos – produtos, resultados e impacto – que se pretende alcançar com um programa/intervenção^11,12^.

A avaliação em saúde tem potencial para apresentar argumentações lógicas de como e porque um programa está ou não atendendo as necessidades específicas para as quais foi criado. Por sua vez, o modelo lógico se apresenta como uma ferramenta avaliativa por detalhar características de um programa, estabelecendo a relação lógica entre seus componentes e os resultados esperados em curto, médio e longo prazo^12^. Modelizar uma intervenção possibilita explicitar os vínculos entre a intervenção e seus efeitos, por meio de uma representação esquemática que revele sua estrutura, processos e resultados. A modelização deve, portanto, explicitar o caminho lógico da intervenção e revelar seus objetivos e, por esse motivo, deve ser feita em interação com os atores que a operam, tornando possível o aprimoramento da inteligibilidade de um sistema complexo^13^. Entre as vantagens na utilização do modelo lógico, estão: a construção de um entendimento comum do programa e das expectativas de recursos, a quantidade de clientes alcançados e seus resultados; a sua utilidade para o desenho ou melhoria do programa; a apresentação do espaço ocupado pelo programa na organização, e a apresentação de um conjunto equilibrado de pontos-chave de medição de desempenho e questões de avaliação, que melhoram a coleta de dados e a utilidade do programa^12^.

Desse modo, os objetivos deste estudo foram elaborar e validar o modelo lógico da linha de cuidado do portador de DRC.

## MÉTODOS

Para a construção e validação do modelo lógico, realizou-se um estudo de caráter descritivo e de abordagem qualitativa, de maio a setembro de 2019, na Região de Saúde do Aquífero Guarani – que comporta 10 municípios, com um total de 945.738 habitantes –, pertencente ao Departamento Regional de Saúde (DRS) 13 – Ribeirão Preto-SP^14^. A linha de cuidado da pessoa com DRC para essa Região de Saúde foi elaborada por um grupo de trabalho multiprofissional e representativo de todos os pontos da RAS, mas seu monitoramento ainda não havia sido iniciado. Houve, então, uma manifestação do DRS 13 aos pesquisadores sobre o interesse em realizar uma investigação que auxiliasse o processo avaliativo, considerando os índices preocupantes dessa Região acerca da prevalência da DRC. Por fim, essa condição justificou a escolha desse campo de estudo.

O modelo lógico foi construído segundo as cinco etapas propostas por McLaughlin e Jordan^12^: 1) Coleta de informações relevantes; 2) Descrição do problema e o contexto; 3) Definição dos elementos do modelo lógico; 4) Construção do modelo lógico; e 5) Validação do modelo lógico.

No desenvolvimento das etapas 1, 2, 3 e 4, foi realizada a pesquisa documental. Os critérios empregados para seleção dos documentos foram: 1) autenticidade, ou seja, o documento ser de origem inquestionável; 2) credibilidade, trata-se de documento original e sem distorções; 3) representatividade, dentro de sua tipologia – no caso, documentos legais –, que apresentam o conteúdo a ser analisado; e 4) significação, ou seja, se o documento era claro e compreensível^15^.

Nas etapas 1 e 2, a pesquisa documental buscou compreender a teoria e a legislação nos âmbitos federal, estadual e regional do SUS, que fundamentaram a elaboração da linha de cuidado da pessoa com DRC; o levantamento de informações sobre a estimativa do número de casos nos diferentes estágios da DRC – estimativa populacional do IBGE –, os parâmetros da Portaria GM/MS nº 1.631/GM/2015^16^, e a identificação da taxa de prevalência de pacientes em diálise na Região de Saúde do Aquífero Guarani. Nessa etapa, também foram consideradas as informações primárias obtidas junto aos entrevistados (Quadro).

**Quadro:** Roteiro da entrevista semiestruturada realizada com informantes-chave para identificação de fatores relevantes de contexto e validação do Modelo Lógico.

Roteiro de perguntas norteadoras	
Identificação sobre contexto da elaboração e implantação da LC da pessoa com DRC	1) Quando foi iniciada a construção da LC da pessoa com DRC? O que determinou o início desse trabalho? 2) Quem são os atores que participaram desse processo? Como aconteceu? 3) Quais os passos posteriores à elaboração da LC? 4) Após a elaboração e demais etapas para a aprovação da LC, quais ações foram disparadas? 5) Quais os principais fatores que determinam a efetiva implantação da LC na sua opinião? Por quê? 6) Como foi realizada a seleção de indicadores para monitoramento e avaliação da LC? Onde os dados para tais indicadores são obtidos? 8) Quem é o grupo condutor regional? Foi realizada a elaboração de ficha de acompanhamento, para monitorar o cumprimento dos protocolos de acompanhamento dos pacientes previstos na LC?
Validação do modelo lógico^12^	1) Na análise do modelo lógico elaborado, a partir de documentos analisados, quais observações você tem a fazer? 2) O nível de detalhamento é suficiente para criar entendimentos dos elementos e suas interrelações? 3) A lógica do programa está completa? 4) A lógica do programa está teoricamente consistente, ou seja, todos os elementos ajustam-se logicamente? 5) Há outros caminhos plausíveis para alcançar os resultados do programa?

LC: linha de cuidado; DRC: doença renal crônica;

No desenvolvimento das etapas 3 e 4, os documentos analisados foram: a PNAPDR^7^, as diretrizes para a organização da RAS e para o Cuidado ao Paciente com DRC no SUS^17,18^, os critérios para a organização da linha de cuidado da pessoa com DRC^9^, o Guia Instrutivo para organização local da linha de cuidado da pessoa com DRC^19^, a normativa sobre o cuidado da pessoa com DRC^20^, e a linha de cuidado da pessoa com DRC da RAS das pessoas com doenças crônicas do DRS 13. As informações sobre essa linha de cuidado foram organizadas no modelo lógico, segundo as dimensões assistenciais – APS, atenção especializada e atenção de alta complexidade –, na forma de um diagrama em cores. Cada cor representou, nas linhas, as dimensões assistenciais e, nas colunas, os componentes da intervenção “linha de cuidado”: insumos/recursos, ações/atividades, produto, resultado intermediário e resultado/impacto final.

O modelo inicial foi apreciado por três profissionais da saúde: uma médica nefrologista, mestre em Ciências da Saúde e doutoranda em Saúde Pública, e duas enfermeiras atuantes na APS, uma mestre e a outra doutora em Saúde Pública. Essas profissionais, escolhidas intencionalmente, contribuíram na checagem dos componentes do modelo lógico e na complementação de informações, caracterizando-se como uma análise preliminar. Após isso, iniciou-se a etapa 5, realizada presencialmente por uma das pesquisadoras e que objetivou apresentar aos informantes-chave os elementos do modelo lógico, seus conceitos e sua finalidade – antes do processo de validação –, e, posteriormente, verificar se os componentes e a organização propostos representavam a lógica da linha de cuidado da pessoa com DRC.

O modelo lógico foi validado por seis informantes-chave, selecionados de forma intencional: um membro da equipe de planejamento de um município do DRS 13, um articulador da APS da Região de Saúde do Aquífero Guarani, um enfermeiro de um serviço de diálise, dois profissionais da equipe de gestão/planejamento do DRS 13 – sendo estes profissionais que participaram da elaboração da linha de cuidado da pessoa com DRC do DRS13 –, e um profissional da equipe de gestão/planejamento da Secretaria de Estado da Saúde de São Paulo (SES-SP), também coordenador do grupo condutor de doenças crônicas não transmissíveis (DCNT). Enfim, realizou-se uma entrevista semiestruturada com os informantes-chave, seguindo os questionamentos apontados por MClauglin e Jordan^12^ (Quadro), e o estudo foi aprovado por Comitê de Ética em Pesquisa (CAAE: 58545116.3.000.5414).

## RESULTADOS

### Caracterização do Contexto

A DRC é reconhecidamente uma doença complexa que exige múltiplas abordagens em seu tratamento. Está associada, inclusive, a altos coeficientes de morbidade e mortalidade, a taxas de prevalência e incidência ainda desconhecidas em muitos países, e a um grande impacto socioeconômico, tornando-se um desafio para a saúde pública mundial^21^. No Brasil, segundo a Pesquisa Nacional de Saúde (PNS, 2013), a prevalência de DRC autorreferida é de 1,42%, ou seja, aproximadamente dois milhões de indivíduos, o que revela a dimensão da doença no país^21^. O diagnóstico precoce, o encaminhamento imediato e a instituição de medidas para diminuir/interromper a progressão da DRC estão entre as estratégias-chave para melhorar os seus desfechos^21^.

A PNAPDR propôs a organização da linha de cuidado integral – promoção, prevenção, tratamento e recuperação –, perpassando todos os pontos de atenção, como a ampliação da cobertura de atendimento aos portadores de hipertensão arterial e de diabetes mellitus, a qualificação da assistência e a promoção da educação permanente dos profissionais da saúde^7^.

Em 2010, foi publicada a portaria que estabeleceu as diretrizes para a organização da RAS no SUS; nela, objetivou-se promover a integração sistêmica de ações e serviços de saúde, com provisão de atenção contínua, integral, de qualidade, responsável e humanizada, além de incrementar o desempenho do SUS, em termos de acesso, equidade, eficácia clínica e sanitária e eficiência econômica^17^. Essa normativa ressaltou que, embora fossem representativos os avanços, ainda era evidente a fragmentação das ações de saúde e a necessidade de qualificar a gestão do cuidado. Assim, o desenvolvimento das RAS se apresentou como um processo organizativo inovador, com potencial para impactar positivamente os indicadores de saúde.

Nessa lógica, nos anos de 2013 e 2014, foi instituída a RAS das Pessoas com DCNT, que estabeleceu diretrizes para a organização de suas Linhas de Cuidado, para os seus princípios e objetivos e para as competências de cada ente federado. As Linhas de Cuidado devem expressar os fluxos assistenciais que devem ser garantidos ao usuário, a fim de atender às necessidades de saúde relacionadas a uma condição crônica e definir as ações e os serviços que serão ofertados por cada componente da RAS das Pessoas com DCNT, baseadas em orientações clínicas e na realidade de cada Região de Saúde^8^.

Por sua vez, os critérios para organização da linha de cuidado da pessoa com DRC, e as diretrizes clínicas para o cuidado, foram publicados em 2014, com a definição das atribuições da APS, da atenção especializada ambulatorial e da assistência de alta complexidade^18^.

A partir da publicação da portaria da linha de cuidado da pessoa com DRC, as regiões de saúde iniciaram o processo de discussão regional sobre ela. De acordo com os informantes-chave, formou-se um grupo condutor com representantes das três regiões de saúde do território do DRS 13, dos prestadores dos serviços de hemodiálise e do grupo de planejamento do DRS 13. O grupo se reuniu para elaborar a linha de cuidado a partir da organização dos serviços, a qual foi aprovada pelas Comissões Intergestores Regionais (CIR) das três regiões do DRS 13 e pela Comissão Intergestores Bipartite (Deliberação CIB/SP – 47/2015). No entanto, segundo os entrevistados, até a data da entrevista, em maio de 2019, não havia sido realizado nenhum monitoramento da linha de cuidado no âmbito da APS, enquanto que os indicadores de qualidade dos serviços de média e alta complexidade eram acompanhados, uma vez que estavam relacionados com a habilitação e remuneração dos serviços especializados^18^.

*“Foi formado um grupo condutor regional com participantes dos municípios, representantes da gestão e dos serviços, aprovados em CIR, e seguimos as etapas previstas na portaria … A Linha de Cuidado foi aprovada na CIR, aguardamos a publicação pelo Ministério da Saúde que, devido a liberação de recursos financeiros, demorou mais de dois anos”*. (Entrevistado n.º 1)

O número de casos estimados nos diferentes estágios de DRC na Região de Saúde do Aquífero Guarani (Tabela 1) indica que aproximadamente 79.070 pessoas – 8,36% da população total – deveriam estar em acompanhamento nos serviços de APS nos estágios 1, 2 ou 3 da DRC, enquanto 1.336 pessoas – 0,14% da população total – seriam acompanhadas em serviços especializados nos estágios 4 ou 5, com ou sem diálise. A prevalência de pacientes em diálise, nessa Região, tem sido crescente ao longo dos anos (Tabela 2). A dificuldade no acesso aos dados do Sistemas de Informação em Saúde (SIS), para confirmar essas estimativas, aponta a um dos nós críticos da gestão em saúde e, principalmente, àqueles referentes aos cuidados prestados na APS. Sendo assim, a ausência de informações confiáveis e tempestivas obstaculiza o monitoramento da linha de cuidado e do planejamento da atenção.

**Tabela 1:** Estimativa do número de casos nos diferentes estágios da doença renal crônica, com base na estimativa populacional do IBGE 2020 e nos parâmetros da Portaria GM/MS nº. 1.631/GM/2015, RS Aquífero Guarani, RP-SP, 2020.

Município	Pop. IBGE 2020	Estimativa IBGE 2020/DSUS acima de 20 anos	Estágio 1 (9,6% pop. > 20 anos)	Estágio 2 (0,9% pop. > 20 anos)	Estágio 3 (1,5% pop. > 20 anos)	Estágio 4 (0,1% pop. >20 anos)	Estágio 5 Diálise novos (0,014% pop. > 20 anos)	Estágio 5 Diálise prevalência (0,075% pop. > 20 anos)	Óbitos (0,013% pop. > 20 anos)
Cravinhos	35.579	26.083	2.504	23	391	26	4	20	3
Guatapará	7.709	5.482	526	5	82	5	1	4	1
Jardinópolis	44.970	32.018	3.074	28	480	32	4	24	4
Luís Antônio	15.292	10.644	1.022	9	160	11	1	8	1
Ribeirão Preto	711.825	535.826	51.439	463	8.037	536	75	402	70
Santa Rita do Passa Quatro	27.600	21.785	2.091	19	327	22	3	16	3
Santa Rosa de Viterbo	26.753	20.190	1.938	17	303	20	3	15	3
São Simão	15.385	11.434	1.098	10	172	11	2	9	1
Serra Azul	14.981	11.819	1.135	10	177	12	2	9	2
Serrana	45.644	31.553	3.029	27	473	32	4	24	4
**Total**	**945.738**	**706.834**	**67.856**	**611**	**10.603**	**707**	**99**	**530**	**92**

IBGE: Instituto Brasileiro de Geografia e Estatística; Datasus: Departamento de Informática do Sistema Único de Saúde.

**Tabela 2:** Taxa de prevalência (por 100 mil habitantes) de pacientes em diálise na Região de Saúde do Aquífero Guarani, por município, 2010 a 2017.

Região de saúde/Mun.	2010	2011	2012	2013	2014	2015	2016	2017
Cravinhos	75,8	71,99	68,25	86,09	60,94	72,48	80,91	83,25
Guatapará	129,29	128,41	99,19	112,58	83,86	97,17	96,55	95,93
Jardinópolis	47,88	47,17	51,64	68,69	67,68	74,09	75,68	60,33
Luís Antônio	62,26	51,9	117,79	90	79,58	92,87	129,17	126,81
Ribeirão Preto	67,57	68,43	69,4	71,29	69,82	74,55	77,11	80,06
Santa Rita do Passa Quatro	67,99	52,89	52,91	64,26	68,06	86,98	87,02	87,06
Santa Rosa de Viterbo	75,5	95,74	99,15	106,6	134,27	125,18	128,36	95,63
São Simão	48,81	69,5	103,89	89,74	116,94	95,98	109,3	74,89
Serra Azul	26,75	26,49	26,24	26	68,67	59,51	75,87	91,93
Serrana	48,94	73,61	67,54	61,62	55,87	69,41	82,75	79,4
**Total**	**65,89**	**67,84**	**69,73**	**72,39**	**71,56**	**76,66**	**80,71**	**80,8**

Fonte: Tabnet, Secretaria de Estado da Saúde de São Paulo (SES/SP). Pacientes atendidos: SES/SP/SIA-SUS - Sistema de Informações Ambulatoriais do SUS. População: Estimativas - Fundação SEADE. 1.Taxa de prevalência: N.º de pacientes submetidos a tratamento de diálise renal no SUS (diálise peritoneal ambulatorial contínua-DPAC, diálise peritoneal automática-DPA, diálise peritoneal intermitente - DPI, hemodiálise, hemodiálise em paciente com vírus da imunodeficiência humana), por 100 mil habitantes na população residente.

*“Hoje o que você tem nos sistemas oficiais, por exemplo, os exames realizados, você consegue puxar, no que a Linha de Cuidado propõe, os exames de cada fase que temos que acompanhar. Então, como produção, isso dá pra levantar. O que é mais complicado, eu acho, é exatamente em relação à Atenção Básica, quantos pacientes eu estou acompanhando, quantos hipertensos tem, qual o estágio deles…”*. (Entrevistado n.º 6)

*“Esses hipertensos é uma dificuldade, a gente não tem ferramenta para avaliar isso. O Hiperdia não tem mais. Ele era bom quando existia, né, e os municípios usavam ele e estava vinculado à distribuição de medicamento…”*. (Entrevistado n.º 2)

Quando perguntados sobre os fatores que consideravam influenciar a efetiva implantação da linha de cuidado, os entrevistados apontaram condições relacionadas ao processo de trabalho, aos modelos de atenção, à qualificação profissional e à qualidade da APS.

*“Os fatores que dificultam são os processos de trabalho, não entendem (os profissionais) o que é rede, cada um faz um pedacinho. Também falta entender de fato o porquê tem tanto paciente. Quando fomos fazer a Linha de Cuidado, não localizamos muitos dados, os municípios não têm esses dados, foi preciso usar referências de estudos”*. (Entrevistado n.º 1)

*“Sou crítico em relação à atuação. Acho que temos as estruturas adequadas, estamos em região privilegiada em relação a suporte e tecnologia, mas pecamos muito na forma como se organiza, modelo de assistência, treinamento e capacitação dos profissionais, acho que isso atrapalha”*. (Entrevistado n.º 2)

*“A especializada e a alta funcionam bem, eles fazem o papel deles, eu acho que tem que intervir é na Atenção Básica”*. (Entrevistado n.º 1)

### Elaboração e Validação do Modelo Lógico

O modelo lógico, construído por meio de um diagrama (Figura), apresenta as atividades e os resultados previstos por dimensão assistencial da RAS, além de identificar os seus insumos e recursos necessários. A definição de cada dimensão, apresentadas no modelo, teve como base os critérios para a organização da linha de cuidado da pessoa com DRC^9^, que subdividem as diretrizes e responsabilidades de acordo com o nível de densidade tecnológica dos serviços da RAS. Da mesma forma, as ações que devem ser desempenhadas fundamentaram-se nas atividades já previstas pelas normas do SUS, sendo, a partir delas, estabelecidos os insumos para a realização do planejamento como um todo.O modelo lógico foi aprimorado a partir de sua apreciação prévia, pela qual informações foram complementadas e verificadas antes da validação. Durante a validação, o modelo foi apresentado a seis informantes-chave, que participaram da elaboração da linha de cuidado da pessoa com DRC ou atuaram em algum ponto da RAS. O questionário semiestruturado (Quadro), proposto por McLaughlin e Jordan^12^, foi aplicado aos participantes na etapa 5, que responderam afirmativamente às questões. Os informantes-chave explicitaram o contexto da elaboração da linha de cuidado e validaram o modelo^12^, não sugerindo a inclusão de nenhuma nova informação.

**Figura:** Modelo lógico. DRC: doença renal crônica; TRS: terapia renal substitutiva; APS: atenção primária à saúde; Kt/V: método que avalia a adequação da diálise do paciente.

## DISCUSSÃO

A DRC tem recebido atenção da comunidade científica internacional e nacional, e sua elevada prevalência está demonstrada em estudos recentes^23^, assim como o aumento na taxa de incidência estimada, que, em 2018, foi 20% superior à observada em 2013. No Brasil, a hipertensão arterial se mantém como a principal causa-base da DRC, seguida pela doença renal do diabetes^24^.

O SUS conta com a PNAPDR desde 2004, mas só quase dez anos depois, em 2013, que se publicaram a linha de cuidado e as diretrizes clínicas da DRC. No entanto, os dados do Censo Brasileiro de Diálise, até 2018, apontaram aumento crescente nas taxas de incidência e prevalência de pacientes em diálise^24^, indicando a necessidade de fortalecimento de políticas de prevenção a esse agravo.

A linha de cuidado da pessoa com DRC estabelece as atribuições dos pontos de atenção da RAS, norteando a organização do trabalho dos profissionais e dos serviços. No entanto, o seu monitoramento e avaliação abrangem basicamente indicadores voltados à alta complexidade^19^, desconsiderando à APS.

Nesse estudo, a elaboração do modelo lógico, a partir da análise de documentos oficiais possibilitou a construção de um primeiro diagrama de relação entre estrutura, processo e resultado. A entrevista com profissionais que atuavam em diversos pontos da linha de cuidado detalhou os componentes desse modelo, tornando-o mais completo e possibilitando a identificação de fatores do contexto interno que poderiam interferir na sua implantação.

Com relação às dimensões assistenciais do modelo lógico, destaca-se a atenção dada pelos informantes-chave à APS. Todos comentaram sobre o seu papel fundamental na organização da linha de cuidado, mas também sobre as suas fragilidades relacionadas ao processo de trabalho, capacitação, qualificação das equipes e à falta de informações para o monitoramento e avaliação.

O primeiro bloco de atividades da dimensão da APS abarca grande parte das ações previstas pela Política Nacional de Atenção Básica^25^. Nesse sentido, a territorialização é a primeira etapa do planejamento. Por meio dela, realiza-se o reconhecimento da área de atuação do serviço de saúde e suas condições socioambientais, caracteriza-se a população e seus problemas de saúde, a dinâmica de sua interação com equipamentos sociais adjacentes e com outros setores, para identificar necessidades e propor ações promotoras e de proteção à saúde, além de prevenção dos agravos^25^.

Portanto, é essencial garantir no território equipes multiprofissionais e infraestrutura que permitam o conhecimento dos problemas/necessidades e potencialidades da comunidade, condições fundamentais para subsidiar o planejamento, monitoramento e avaliação das ações de saúde.

No segundo bloco, cujo insumo é a realização de atividades educativas, a ausência da educação popular em saúde como ação relaciona-se ao fato de ela não constar no item “educação em saúde”, das normativas consultadas e da vivência dos informantes-chave sobre o tema. Todavia, entendemos que seja importante para o empoderamento do usuário e consequente participação na tomada de decisão.

Os blocos seguintes se relacionam com a garantia de atendimentos na atenção especializada, transporte e sistema de referência e contrarreferência. Sobre o uso do prontuário eletrônico, embora pudesse ser considerado como um insumo, diante da disponibilidade do PEC e-SUS pelo Ministério da Saúde, foi considerado como uma ação, seja o próprio PEC e-SUS ou qualquer outro sistema do tipo. O prontuário eletrônico permite a otimização dos atendimentos clínicos, a acessibilidade aos dados da assistência e dos procedimentos realizados, a integração de informações, o cadastramento dos domicílios, além de apoiar o ensino e a pesquisa. Ademais, esse prontuário favorece a comunicação na RAS, por exemplo, por meio de um sistema de referência e contrarreferência, contribuindo para a coordenação e continuidade do cuidado e vinculação do usuário às equipes.

Em relação aos produtos da APS, embora sejam difíceis de serem mensurados, referem-se ao que se espera das atividades propostas. Acredita-se que o modelo lógico desperte reflexões sobre eles e, consequentemente, a eleição de indicadores de estrutura, de processo e de resultados por dimensão da linha de cuidado.

A dimensão da atenção especializada foi referida como a parte da linha de cuidado que “mais funciona”, com indicadores de monitoramento bem definidos, assim como a alta complexidade. Destaca-se que, entre os entrevistados, não estavam profissionais especialistas da alta complexidade, o que pode ser uma limitação do estudo, considerando que estes poderiam propor modificações e ou complementações ao modelo.

O modelo lógico, embora tenha sido elaborado em uma RS, o que pode representar uma limitação, comporta as três dimensões da atenção à saúde: primária, secundária e terciária. A sua utilização por profissionais e gestores, cotejada com as distintas realidades, pode colaborar com o monitoramento/avaliação da linha de cuidado, e com a identificação de potencialidades e desafios, auxiliando no planejamento e na tomada de decisões^26^.

## CONSIDERAÇÃO FINAL

Há o reconhecimento de que, no país, a abordagem ao portador de DRC no SUS necessita de incremento. A linha de cuidado da pessoa com DRC, ao detalhar as atribuições pelas dimensões de atenção da RAS, caracteriza-se como um norteador da organização do processo de trabalho dos profissionais e dos serviços, com ênfase na APS, componente ainda pouco explorado e valorizado.

No contexto da avaliação em saúde, o modelo lógico apresentado auxilia na proposição de indicadores de desempenho aplicáveis às três dimensões assistenciais, mas principalmente à APS, pois, embora seja reconhecida como ordenadora da rede e coordenadora do cuidado, apresenta várias debilidades organizacionais, e, por isso, tem sido desprivilegiada nas discussões e nos investimentos. Ainda, os indicadores a serem elaborados a partir dessa linha de cuidado da pessoa portadora de DCNT podem ser empregados em outras Linhas do tipo, contribuindo com o aperfeiçoamento da ação gestora e viabilizando a efetiva implementação dessa estratégia de organização do cuidado.

Admite-se que o modelo lógico sugerido tenha potencial de contribuir com processos de avaliação da linha de cuidado da pessoa com DRC, com vistas ao alcance de melhores resultados no manejo dessa doença, tanto para o seu portador quanto para o sistema de saúde.
